# The Role of Multimodality Imaging Approach in Acute Aortic Syndromes: Diagnosis, Complications, and Clinical Management

**DOI:** 10.3390/diagnostics13040650

**Published:** 2023-02-09

**Authors:** Francesco Perone, Marco Guglielmo, Michele Coceani, Lucia La Mura, Ilaria Dentamaro, Jolanda Sabatino, Alessia Gimelli

**Affiliations:** 1 Cardiac Rehabilitation Unit, Rehabilitation Clinic “Villa delle Magnolie”, Castel Morrone, 81020 Caserta, Italy; 2 Department of Cardiology, Division of Heart and Lungs, Utrecht University Medical Center, 3584 CX Utrecht, The Netherlands; 3 Diagnostic and Interventional Cardiology Unit, Fondazione Toscana Gabriele Monasterio, 56124 Pisa, Italy; 4 Department of Advanced Biomedical Sciences, University Federico II of Naples, 80133 Naples, Italy; 5 Cardiology Department and Cardiovascular Imaging Laboratory, Hospital Miulli, 70124 Bari, Italy; 6 Pediatric and Congenital Cardiology Unit, Department for Women’s and Children’s Health, University Hospital of Padova, 35128 Padova, Italy; 7 Imaging Department, Fondazione Toscana Gabriele Monasterio, 56124 Pisa, Italy

**Keywords:** acute aortic syndromes, multimodality imaging, echocardiography, computed tomography, magnetic resonance imaging, aortography

## Abstract

Acute aortic syndromes are life-threatening conditions with high morbidity and mortality. The principal pathological feature is acute wall damage with possible evolution towards aortic rupture. Accurate and timely diagnosis is mandatory to avoid catastrophic consequences. Indeed, misdiagnosis with other conditions mimicking acute aortic syndromes is associated with premature death. In this view, cardiovascular imaging is necessary for the correct diagnosis and management. Echocardiography, computed tomography, magnetic resonance imaging, and aortography allow for diagnosis, guarantee immediate treatment, and detect associated complications. Multimodality imaging is essential in the diagnostic work-up to confirm or rule out acute aortic syndromes. The aim of this review is to highlight the contemporary evidence on the role of single cardiovascular imaging techniques and multimodality imaging in the diagnosis and management of acute aortic syndromes.

## 1. Introduction

Acute aortic syndromes (AAS) represent a spectrum of interrelated disorders characterized by the disruption of the aortic integrity, and are associated with high morbidity and mortality. These conditions include aortic dissection (AD), accounting for the majority of AAS (80%), intramural hematoma (IMH, ~15%), and penetrating aortic ulcer (PAU, ~5%) [1,2,3].

AD is described as a separation of the aortic wall layers caused by an intimo-medial tear, resulting in the creation of a false lumen that propagates within the medial layer. Thus, AD typically shows the appearance of a dissection flap, an entry tear, and two aortic channels (a true and false lumen). No modern classifications have substituted the older Stanford and DeBakey ones that remain the most commonly used. AD involving the ascending aorta is defined as a Stanford type A dissection, regardless of distal extension; when the disruption is distal to the left subclavian origin, on the contrary, it is considered to be type B. The DeBakey classification recognizes types I, II, and, III, with type I affecting both the ascending and descending aorta, type II the ascending aorta and the arch only, and type III involving the descending aorta but sparing the arch and the ascending aorta. The Stanford classification is used to guide different treatment options. Indeed, surgery is recommended in type A AD, while medical therapy is suggested in uncomplicated type B AD. IMH is instead a haematoma created within the media layer and secondary to the rupture of the vasa vasorum. Finally, PAU is an ulceration of an atherosclerotic aortic plaque penetrating towards the internal elastic lamina and into the media [4].

**Figure 1:**
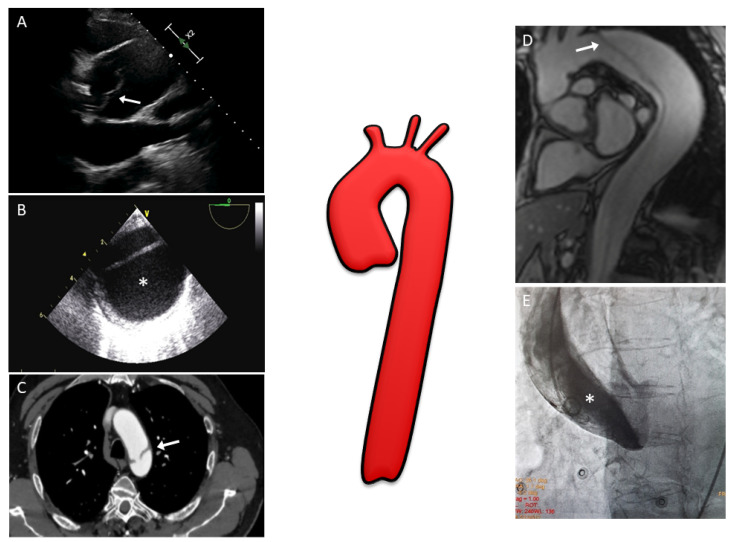
Multimodality imaging assessment of aortic dissection: (**A**) Two-dimensional transthoracic echocardiography showing a linear echo of an intimal flap (arrow) in a dilated aortic root above aortic valve level; (**B**) Two-dimensional transesophageal echocardiography in patients with aortic dissection involving the entire aorta; the false lumen (*) is typically larger and often compresses the true lumen, potentially affecting distal aortic flow; (**C**) CT image with evidence of the intimal tear (arrow) at the level of the aortic arch; (**D**) MRI with SSFP imaging in the oblique sagittal plane showing an intimal flap (arrow) from the aortic arch to abdominal aorta; (**E**) Aortic angiography performed in a patient with suspected inferior ST-segment elevation myocardial infarction revealing a type A aortic dissection (one may note that the pigtail catheter is located in the false lumen (*) of the dissection). CT, computed tomography; MRI, magnetic resonance imaging; SSFP, steady-state free precession.

The advent of multi-modality imaging in the assessment and management of AAS has multiple applications, not only for diagnosis, but also for risk stratification and planning of the acute intervention, as well as for the evaluation of potential complications and/or the candidacy for intervention or re-intervention in the subacute or chronic phases (Figure 1).

In this document, our aim is to critically review the distinctive multi-modality imaging features of AAS, while at the same time providing an update and overview of diagnostic strategies and the current management of these disorders.

## 2. Aortic Dissection

### 2.1. Transthoracic and Transesophageal Echocardiography

Echocardiography is renowned for being a non-invasive, safe, and accurate imaging modality used in the clinical evaluation of cardiovascular disease and plays an important role in the diagnosis of aortic diseases. Transthoracic echocardiography (TTE) can be performed at the patient’s bedside, as well as in the emergency and critical care units, and for this reason the European Society of Cardiology guidelines [5] recommend it in patients who are clinically suspected of having an AD (class I, level of evidence C). 

The diagnosis of AD by TTE is based on detecting intimal flaps in the aorta. The sensitivity and specificity of TTE range from 77–80% and 93–96%, respectively, for ascending AD, and varies according to the use of contrast agents [6]. Nevertheless, TTE is successful in detecting the distal dissection of the thoracic aorta in only 70% of patients [7]. On transthoracic M-mode echocardiography, floating intimal flaps, the enlargement of the aortic root, and an increase in the aortic wall thickness were considered signs of AD. With the introduction of two-dimensional (2D) echocardiography and the feasibility of taking suprasternal, subcostal, and substernal views, it has become possible to directly visualize the presence of floating intimal membranes, intimal tears, and false lumens on the ascending aorta and aortic arch [8]. Thanks to the superior axial image resolution offered by parasternal long-axis views, mobile dissection membranes within the ascending aorta can be seen in AD patients with optimal windows (Figure 1A). The parasternal short-axis view is useful to evaluate the morphology of the aortic valve and location of any aortic regurgitation. Aortic regurgitation is a frequent finding in patients with type A AD, occurring in 40–76% of patients [9]. TTE can also offer immediate evidence of other complications which can accompany an AD, such as cardiac tamponade, severe aortic dilation, regional wall motion abnormalities, and severe left ventricular systolic dysfunction, all of which may influence surgical management [10]. Since sub-optimal image quality can be a limitation in some patients, an AD cannot always be completely ruled out by TTE alone, and further imaging such as transesophageal echocardiography (TOE) or computed tomography (CT) of the aorta should be considered if clinical suspicion remains high [11].

TOE is currently considered as one of the reference techniques in AD diagnosis, with a very high diagnostic accuracy for the detection of both ascending and descending aortic diseases (Figure 1B) [12]. Indeed, the proximity of the oesophagus and the thoracic aorta permits a high-resolution image. Furthermore, the availability of multiplane imaging allows incremental assessment of the aorta from its root to the descending aorta. Several studies have demonstrated the accuracy of TOE in the diagnosis of AD with a sensitivity of 86–100%, a specificity of 90–100%, and a negative predictive value of 86–100% [13]. Owing to the interposition of the right bronchus and trachea, a short segment of the distal ascending aorta, just before the innominate artery, remains a ‘blind spot’ [5]; Evangelista et al. demonstrated that contrast-enhanced echocardiography can solve this problem [12]. One of the main limitations of TOE is the presence of ultrasound artefacts in the ascending aorta which are common, particularly when dilated [14]; in this case, M-mode tracings differentiate between intimal flap and imaging reverberations [15]. TOE may also identify the presence of leaks and/or small re-entry tears with much higher sensitivity than angiography. This is also important for the prognosis of patients with residual patent false lumen in the descending aorta and the presence of a large proximal entry tear (>10 mm) defined by TOE, which indicates a high risk of mortality and the need for surgical or endovascular treatment during the follow-up [16]. Other limitations of this technique could concern the discomfort for patients, with the possible need for sedation, and the intra- and inter-operator variability.

### 2.2. Computed Tomography

Thanks to its high spatial resolution, full assessment of thoracoabdominal aorta, short acquisition time, and wide availability, CT represents the ideal technique for diagnosing AAS.

The typical scan protocol first includes a non-contrast CT followed by an arterial acquisition [17]. A late thoracoabdominal scan can be added to improve the detection of visceral malperfusion in the acute setting of AD and distinguish slow flow from thrombus in the false lumen [18]. Indeed, this late (venous) phase is often useful to confirm the circulation of the false lumen, which may not be opacified in the arterial phase. Electrocardiogram (ECG) gating should always be used to avoid motion artifacts that can be misinterpreted as intimal flaps. The entire aorta must be assessed to determine the distal extent of the dissection and to identify the ischemia of the abdominal organs, which have important prognostic implications. In the case of a cerebrovascular accident, a CT head scan is mandatory. Dedicated injection protocols are used, considering the speed of scan acquisition and coverage to optimize image quality and reduce the volume of contrast agents [19]. The main limitations concern the use of iodinated contrast and ionizing radiations.

The sensitivity and specificity of CT for AD is approximately 98–100% [20], and it represents the modality of choice for dissection in the majority of patients [21]. CT can differentiate between type A and B AD, helping to localize intimal entry site, involvement, relationship with the false or true lumen of the branch vessels, and organ ischemia (Figure 1C) [19]. Moreover, coronary ostia involvement can be detected with CT [22].

The classical finding of AD in CT is an intimal flap with a partition between the true and false lumen. Supporting signs are the internal displacement of intimal calcifications, the delayed enhancement of the false lumen, the widening of the aorta, mediastinal, pleural, or pericardial hematoma [23], and signs of cardiac tamponade. The latter is suspected in the presence of a large pericardial effusion, dilatation of superior and inferior vena cava, reflux of contrast agents into the inferior vena cava and azygos vein, compression of the cardiac chambers, and the bowing of the interventricular septum [24]. A thorough clinical evaluation does not always provide a clear understanding of the cause of acute chest pain. For these cases, a triple rule out protocol has been proposed in which coronary arteries, the thoracic aorta, and pulmonary arteries are simultaneously examined with CT. However, triple rule out scans provide greater anatomic coverage than dedicated protocols, which leads to a higher radiation dose. In addition, they require a higher contrast load to opacify both the right and left circulations, as well as more time to report the scan. Multiple studies have failed to demonstrate superior clinical outcomes with triple rule out to justify the increased radiation, contrast dose, and readout time [2]. Apart from its essential crucial diagnostic role, CT is used in patients with AD for planning interventional surgical or percutaneous interventions. Specifically, measurements of vessel diameter, angles, aneurysm neck size, and proximal and distal landing zones for stent grafts are essential in determining thoracic endovascular aortic repair (TEVAR) eligibility [25].

### 2.3. Magnetic Resonance Imaging

Magnetic resonance imaging (MRI) is a highly accurate, noninvasive imaging modality, with a sensitivity and specificity of almost 98% for detecting AD [26]. Additionally, it does not require ionizing radiation or iodinated contrast, making it the most suitable test for patients with impaired renal function, known severe allergies to iodated contrast material, and who are pregnant. The limitations of MRI in acute AD apparently include long imaging time, the need for patient cooperation to avoid motion-related image degradation, and difficulty in monitoring acutely ill patients. Nevertheless, Wang et al. demonstrated that the use of MRI for evaluation of thoracic aortic dissection is well-tolerated by emergency department patients [27], despite the fact that MRI is currently not used in the vast majority of centres to search for acute dissection, it being more useful in the follow-up of chronic type B dissection. Other limitations include the risk of nephrogenic systemic fibrosis with GFR < 30 mL/min/1.73 m^2^ when gadolinium is used, and the poor assessment of arterial wall calcification.

In a suspected case of AD, the standard MRI examination should begin with rapid spin-echo black blood acquisitions covering the aorta to outline aortic shape and diameter and to rule out alterations in wall structure [28]. In the axial plane, the intimal flap is detected as a line inside of the aortic lumen. In stable patients, adjunctive gradient-echo sequences or phase contrast images can be instrumental in identifying aortic regurgitation and entry or re-entry sites as well as in differentiating slow flow from the thrombus in the false lumen [29,30,31,32]. With balanced steady-state free precession (SSFP) techniques, image contrast is determined by T2/T1 ratios, allowing the detection of the intimal media flap, with no gadolinium contrast media [33,34]. These images are typically acquired initially in axial and oblique sagittal projections and gated to the cardiac diastolic phase (Figure 1D). Cine SSFP MRI can be used to further delineate the entry and exit zones of the intimal media flap and the presence of aortic regurgitation thanks to the detection of flow turbulence. Quantitative data on flow velocity and volume, in the true and false lumen, can be obtained from the velocity maps of phase contrast sequences. In addition, contrast-enhanced MRI angiography may be performed quickly (in a few seconds without any need of ECG triggering) and will provide information regarding branch vessel involvement, the presence of intraluminal abnormalities, PAU, and will demonstrate the intimo-medial flap location and the entry and exit tears in the dissection. Despite the advantages, the image quality of dynamic MRI angiography proved to be inferior to high-resolution three-dimensional (3D) MRI [35]. Four-dimensional (4D) flow MRI could be a potential tool in AD patients thanks to flow quantification and hemodynamic information. Unfortunately, so far it has currently been used only in ex vivo studies and in patients with chronic dissection [36,37].

### 2.4. Aortography

In the past, aortography represented the gold standard for the diagnosis of AD [38]. In more recent years, aortography has been almost completely replaced by equally accurate, but less invasive, techniques. Nevertheless, aortography remains the benchmark against which all other imaging modalities should be measured [39]. Furthermore, aortography still maintains an important role in patients who undergo coronary angiography (for example, in stable patients in which concomitant coronary artery disease is suspected). It should also be kept in mind that AAS may mimic an acute coronary syndrome and, as a result, the correct differential diagnosis will be made only at the time of invasive coronary angiography. Finally, aortography is an indispensable guide for endovascular procedures, which are more frequently carried out in patients with type B dissection [5].

Aortography visualizes all features of AD: the intimal flap, true lumen (which is usually compressed), false lumen, craniocaudal extension, indirect visualization of the coronary arteries, possible aortic regurgitation, and aortic rupture (Figure 1E). Various projections may be necessary to evaluate every aspect of aortic anatomy and the potential involvement of aortic side branches [22]. In the case of a type A dissection, aortography permits the assessment of the blood vessels which originate from the aortic arch, thus facilitating surgical planning. However, because aortography is a “lumenogram”, it does not provide any information on aortic wall thickness. Caution must be employed in performing aortography because, if the false lumen is cannulated with the diagnostic catheter, a sudden increase in pressure during contrast injection may lead to aortic rupture. To this end, manual injection may be safer, although less effective in the visualization of the entire dissection, especially in the case of low blood flow. Moreover, in patients with chronic kidney disease, aortography may lead to contrast induced nephropathy. In addition to the use of contrast medium, the invasive nature of the procedure and radiation exposure are other limitations to this technique. The specificity of aortography is high at 95%, but sensitivity may be lower than other techniques due to the inability in certain cases to differentiate the two lumens of the aorta (for example, because of a completely thrombosed false lumen) [4].

## 3. Intramural Hematoma

### 3.1. Transesophageal Echocardiography 

**Figure 2:**
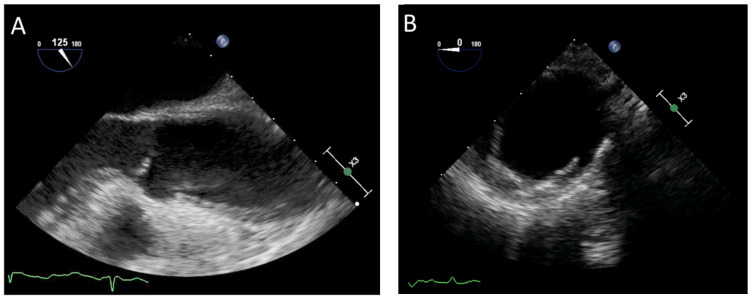
Transesophageal echocardiography assessment in two different patients with acute aortic syndrome: (**A**) intramural hematoma and (**B**) penetrating aortic ulcer.

IMH is classified among AAS and represents about 15% of cases. Specifically, it is defined as Type A when it affects the ascending aorta and the aortic arch and type B when it involves the descending aorta. [5]. Echocardiography is a useful method for diagnosing IMH, preferably with TOE due the low sensitivity of TTE (<40%) [40]. IMH is typically diagnosed in the presence of crescentic or concentric thickening of the aortic wall > 0.5 cm [41]. Furthermore, it is characterized by the absence of intimal tear, mobile dissection flap, and Doppler signal indicating a communication between the aortic lumen and hematoma. The luminal shape is preserved and the luminal wall is curvilinear and smooth. Other secondary findings are the presence of intimal calcification displacement and echo-lucent areas in the aortic wall hematoma (Figure 2A) [2]. Complications due to IMH cover a broad spectrum. Indeed, hematoma can evolve towards classical or localized AD, saccular or fusiform dilatation, pseudoaneurysm, or aortic rupture in the pericardium, pleura, or mediastinum [42]. Limitations in using the TOE in this setting include the limited visualization of all segments of the aorta, semi-invasive nature, and operator dependency. In several situations, diagnosing IMH can be challenging. Indeed, classical AD with thrombosed false lumen may be similar to IMH, but the diameter is generally larger and the circumferential extension is smaller. Instead, aortic atherosclerosis and aortic aneurysm with mural thrombus differ mainly in the presence of an irregular luminal surface. Furthermore, intimal calcification displacement is useful for further differentiation and characterization. Finally, a hemizygous sheath is also considered in the differential diagnosis with IMH. This is a normal structure, a periaortic fat pad situated 30–35 cm from the incisors during TOE [43]. 

### 3.2. Computed Tomography

**Figure 3:**
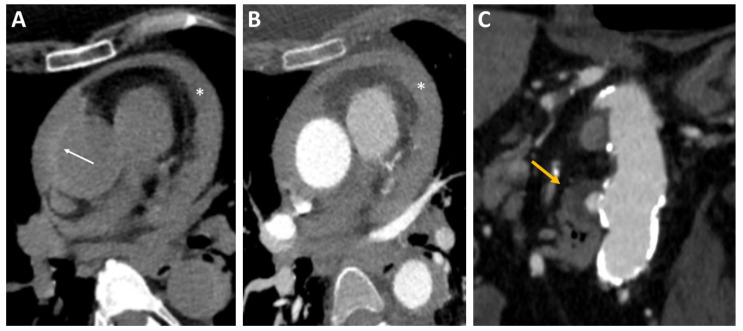
Intramural hematoma on CT using (**A**) non-contrast CT and (**B**) CT angiography: crescentic, high-attenuating regions of eccentrically thickened aortic wall on non-contrast CT (arrow). A diffuse pericardial effusion (*) was also visible in both scans. (**C**) Penetrating ulcer on CT: CT angiography image showing a penetrating ulcer of the descending aorta as a contrast-filled, out-pouching into the thickened aortic wall (arrow). CT, computed tomography.

Non-contrast CT from the supraortic vessels to the aortic carrefour is crucial to diagnosing IMH. IMH is visible on unenhanced CT typically as an eccentric, hyperdense (60–70 HU), crescentic area of thickening of the aortic wall (>0.5 cm), showing no enhancement after administration of intravenous contrast that extends in a longitudinal, non-spiral fashion (Figure 3A,B). The sensitivity and specificity values of CT for IMH are close to 100% [44]. In addition to the well-known limitations of this technique, another limitation is the difficulty in differential diagnosis with aortitis. Type A IMH is at higher risk of evolving towards overt dissection and is associated with the increased incidence of hemopericardium and cardiac tamponade. Management of IMH type A is usually surgical [45].

Other CT findings that can be useful to predict the negative evolution of the IMH include an IMH high mean IMH thickness greater than 10–11 mm, compression of the true aortic lumen, a maximal ascending aortic diameter greater than 50 mm, or descending aorta greater than 45 mm, association with PAU, and the presence of pericardial effusion [46]. These high-risk features can help to decide the management of type B IMH if conservative or interventional using TEVAR.

### 3.3. Magnetic Resonance Imaging

MRI has the unique ability to determine the age of the hematoma based upon T1 and T2 signal characteristics [47,48,49]. Despite this, MRI is currently used as a second level investigation in the acute patient in the presence of diagnostic uncertainty.

**Figure 4:**
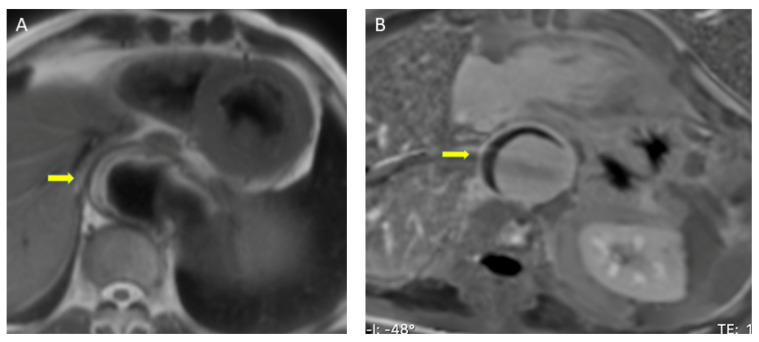
Magnetic resonance imaging assessment of intramural hematoma (arrows). (**A**) Spin-Echo sequence. The eccentric thickening of the aortic wall has a high T1 signal, eliminating the possibility that it is an acute stage. (**B**) Late gadolinium- enhancement sequence. Aortic eccentric wall thickening with no mural enhancement, suggestive of intramural hematoma.

Breath-hold ECG-gated fast spin echo images acquired with T1 and T2 weighting are used to assess aortic calibre, aortic wall thickness, and aortic wall signal change. These are acquired as contiguous transverse sections from the apexes to the infrarenal abdominal aorta and also as sections paralleling the long axis of the aortic arch. On MRI, the spin-echo black blood images of an IMH show a crescent-shaped area of eccentric thickening, and exhibit the expected signal characteristics associated with the transition of hemorrhage from deoxyhemoglobin (low T1 and T2) in the hyperacute stage to intracellular methemoglobin (high T1, intermediate T2) in the subacute stage and extracellular methemoglobin (high T1 and T2) in the late stage of IMH (Figure 4A).

Dynamic SSFP sequences are used to assess aortic valve function and the calibre of the aortic root. These are acquired in both transverse and coronal planes through the left ventricular outflow tract (LVOT). If aortic regurgitation is present, its severity can be quantified via flow sensitive phase contrast sequences. Finally, a gadolinium-enhanced aortic angiography study could be performed. However, the risk of nephrogenic systemic fibrosis with GFR < 30 mL/min/1.73 m^2^ represents a limitation, in addition to the difficulty in monitoring patients in the acute phase.

A small study suggested three magnetic resonance angiography (MRA) parameters to distinguish IMH from type B dissection: (1) no visualized entry tear, (2) no contrast uptake in aortic lesion on first pass angiography, and (3) no contrast uptake in the aortic lesion on the equilibrium phase T1-weighted sequence [50]. In addition, post-gadolinium sequences are useful to discriminate between aortitis and IMH: mural enhancement is not an expected feature of IMH [51,52] (Figure 4B).

### 3.4. Aortography

In the diagnosis of IMH, aortography has a more limited role compared to classic dissection because the intima is intact and, as a result, the lumen of the aorta remains unaffected [2]. The only clue to IMH provided by aortography may be a faint shadow separated from the aortic lumen, but confirmation from other techniques is mandatory. In this context, the accuracy of aortography is reduced and the technique is limited by the use of contrast medium and the invasive nature. Because evolution to classic dissection or aortic rupture is relatively frequent, prompt intervention is frequently indicated. In addition to classical surgical techniques, endovascular procedures may be performed which have the advantage of being less invasive, an aspect of primary importance considering that patients with IMH tend to be older and may have multiple comorbidities. In this context, aortography becomes necessary to confirm the diagnosis and must be performed during all phases of the procedure to aid in the deployment of endovascular stents [5].

## 4. Penetrating Aortic Ulcer

### 4.1. Transesophageal Echocardiography

TOE is among the methods for detecting PAU and evaluating possible complications (Figure 2B). Although it could be situated in any segment of the aorta, PAU is infrequently located in the ascending aorta, aortic arch, and abdominal aorta, while it is more commonly present in the mid and distal segments of the descending aorta [5]. The key diagnostic feature is the presence of a crater-like out-pouching with jagged edges of the aortic wall and multiple irregularities of the intimal wall. Color Doppler can be useful for describing turbulent flow within and at the entrance orifice of the ulcer. This method ensures the excellent visualization of the aortic wall and provides maximum depth of ulcer penetration from the aortic lumen. In addition, the location, width, and length of the ulcer and aortic diameter at the level of the PAU may also be detected [2]. Localized IMH is generally associated with the lesion and could involve the rest of the aorta [53]. The diagnostic value of the TOE is moderate and the use is restricted by the semi-invasive nature. Echocardiography can detect complications associated with PAU. Acute AD, pseudoaneurysm, or rupture of the aorta could result from the propagation of ulcer erosion [54]. Furthermore, PAU can evolve towards the formation of a saccular, fusiform, or false aneurysm. Finally, TOE has the capacity to differentiate PAU from an ulcer-like projection (ULP). Specifically, ULP is a localized pouch with a large communication orifice protruding generally in the IMH.

### 4.2. Computed Tomography

CT today represents the most used technique for the diagnosis and follow-up of PAU thanks to its high spatial resolution, which allows for a detailed assessment of atherosclerotic plaques, calcium, and the aortic wall [28]. 

CT characteristic features of PAU are an aortic out-pouching in the presence of aortic intima calcifications and severe atherosclerotic disease. The edges of the out-pouching are usually irregular or jagged [55] (Figure 3C). PAU can be distinguished with CT from ulcerated aortic plaque. The latter is a minimal disruption of the intima without the involvement of the media layer of the aorta. On CT images, ulcerated plaques appear like a crater within an atherosclerotic plaque which, in contrast to PAU, does not alter the aortic outline [56].

CT imaging can be helpful in stratifying the risk of patients with PAU to select the appropriate management. In particular, patients with PAU diameters over 13–20 mm and more profound than 10 mm and concomitant IMH are associated with a worse prognosis [57]. Choosing this technique, the risk of nephropathy and allergy induced by contrast medium and use of ionizing radiation must be considered.

### 4.3. Magnetic Resonance Imaging

PAU can be well visualized on MRI, as well as the associated IMH. Ulcer craters can be documented on spin-echo images as a signal void within the thickened aortic wall [58]. The immediate arterial phase images on contrast-enhanced MRI may show an outpouching that fills with contrast and allows the evaluation of the size and extent of ulceration; however, the delayed images add enhanced visualization of the aortic adventitia and surrounding soft tissues, thus allowing more definitive characterization of the aortic enlargement and associated structures in penetrating aortic ulceration. 

The diagnostic challenge consists in distinguishing PAU from the ulcerated atherosclerotic plaque and the IMH from the intraluminal thrombus. In 1990 Yucel et al. studied seven patients with acute chest pain and penetrating aortic ulcers by MRI imaging compared with angiography and CT. They demonstrated that MRI, thanks to the ability to image the aortic wall, was superior to angiography in depicting the extent of intramural thrombus and was superior to CT in differentiating acute IMH from atherosclerotic plaque and chronic intraluminal thrombus [59]. As noted for other pathologies, limitations include extended scan time, difficulty in monitoring patients in the acute setting, and risk of nephrogenic systemic fibrosis. In this scenario, the use of MRI can be decisive in the definitive diagnosis of PAU and in recognizing associated aortic diseases. 

### 4.4. Aortography

Compared to AD, patients with PAU are older and present risk factors for atherosclerosis such as arterial hypertension, dyslipidemia, and smoking [60]. Once again, aortography is not a first-line imaging test [2]. In addition, the limitations are related to the invasive nature and the use of the contrast medium and radiation exposure. The typical aspect is that of an irregular extroflection of the aortic lumen similar to a gastric ulcer observed with a barium examination. This type of AAS is often multiple and may vary in size. Any segment of the aorta may be involved, although the ascending aorta is generally spared. In addition, PAU, like IMH, progresses more aggressively compared to classic dissection, and rupture is not infrequent if left untreated. For all of these reasons, endovascular procedures—and consequently aortography—play a key role in the management of PAU [61].

## 5. Choice of Imaging Modality and Clinical Decision

In an emergency scenario, a rapid comprehensive diagnostic work-up is necessary, including clinical assessment (calculation of pre-test probability of disease), laboratory data (D-dimer and troponin amongst others), chest X-ray, and ECG in order to expedite the appropriate aortic imaging studies [62,63,64,65,66]. Diagnostic multimodality imaging in the setting of AAS has several goals: the confirmation of clinical suspicion, classification of the disease, and the evaluation of urgency indicators are in fact mandatory. Clinical pre-test probability is considered higher in the presence of severe and/or abrupt interscapular chest pain associated with signs of aortic regurgitation, occluded aortic side branches causing ischaemia, or pericardial effusion [5,67].

In this setting, bedside TTE combined with additional TOE have the priority in order to exclude new-onset aortic regurgitation, pericardial effusion, or to visualize proximal dissection. Modern ultrasound equipment is in fact mobile and particularly useful at the bedside for unstable AAS and/or for detecting painless AD [68]. TOE evaluation, added to the transthoracic suprasternal view, has good accuracy to diagnose acute AD (type A), even intraoperatively, with near 100% sensitivity and specificity [69]. Color-Doppler is often able to assess entry sites and/or false lumen flow in order to confirm a proximal dissection. However, CT remains the initial diagnostic imaging technique in the patient with suspected acute AD, especially if stable. Instead, in the unstable patient, echocardiography represents a reasonable alternative. Multidetector CT scanning of the entire aorta may be the logical next step, if considered safe. On the other hand, MRI has no place in the acute emergency setting of symptomatic patients remaining more useful in the follow-up.

Given the optimal accuracy of all multi-modality imaging modalities, imaging protocols should be adapted to local expertise and availability, the patient’s clinical condition, and the target of interest (Table 1, Table 2 and Table 3).

CT technology has currently replaced invasive diagnostic angiography for the thoracic and abdominal aorta. CT is rapid, accurate, and has high spatial resolution, but requires patient transportation and, thus, stable haemodynamic conditions [70].

MRI angiography is also capable of providing high-resolution aortic imaging with three–dimensional post-processing; it needs neither iodinated contrast nor ionizing radiation, being particularly useful for patients intolerant to contrast due to allergy or renal failure [71,72,73]. MRI angiography is less affected by calcification than CT and is better suited to depict luminal narrowings or IMH even in the presence of atherosclerotic calcification [74]. However, MRI has lower spatial resolution than CT and metals (implanted stents, clips) may cause distortion and artifacts. In addition, many pacemakers, defibrillators or older mechanical valves are contraindications for MRI.

## 6. Follow-Up

After an AAS, surviving patients require lifelong follow-up. The imaging approach is crucial in prognostic assessment and to detect evolution and complications. The cardiac imaging techniques utilized for surveillance are echocardiography, CT, and MRI. CT is the most commonly performed for the long-term follow-up after an AAS. However, especially in young patients, this technique must consider radiation exposure. MRI is a valid alternative and avoids the use of iodinated contrast in addition to radiation exposure. Furthermore, in the IMH, it identifies the evolution of temporal bleeding and new hemorrhagic episodes. Cardiac imaging follow-up is indicated both in patients managed with medical therapy alone and in those treated surgically or with TEVAR [75,76,77,78,79]. Monitoring is crucial for patients who receive a medical management. Indeed, one-third of those with type B aortic syndromes stabilized medically will ultimately be treated with surgery for aneurysmal degeneration of the dissected segment. In patients receiving TEVAR, CT is the first-choice imaging technique, and it is able to identify endoleaks, aneurysmal degeneration, and disease progression [57].

## 7. Conclusions

A multimodality imaging approach in the AAS is indispensable for accurate and timely diagnosis. In this setting, prompt diagnosis is mandatory considering this life-threatening condition which requires adequate and immediate treatment. In addition, multimodality imaging adds key information about urgency indicators and the associated complications. A correct and high-quality diagnostic work-up improves the poor prognosis in this emergency condition. Advances in cardiovascular imaging techniques have led to a better understanding of the pathophysiology and improvement in diagnosis and management. The appropriate choice of imaging modality is based on the accuracy, advantages, and limitations of the techniques. However, the clinical conditions of the patient and local availability and expertise should be also considered. The correct application of the imaging algorithm and cardiovascular techniques is crucial for the diagnosis and management of AAS.

## Figures and Tables

**Table 1:** Advantages and limitations of imaging techniques in the diagnosis of aortic dissection.

Imaging Technique	Advantages	Limitations
Transthoracic echocardiography	-Frequently used technique for measuring proximal aortic segments in clinical practice -Visualization of the aortic valve and ascending aortic structure in real time at the patient’s bedside or in the emergency and critical units -Rapid identification of any complications such as cardiac tamponade, severe aortic dilation, regional wall motion abnormalities, and severe left ventricular systolic dysfunction	-Restricted in patients with abnormal chest wall configuration, obesity, pulmonary emphysema, and in patients on mechanical ventilation -Low sensibility in detecting distal dissection of the thoracic aorta -Intra- and inter-operator variability
Transesophageal echocardiography	-Useful in the initial diagnosis and follow up of aortic dissection -Very high diagnostic accuracy -Crucial role in the pre-operative, intra-operative, and post-operative control of surgically treated aortic disease	-Presence of a “blind spot” -False positive results could occur because of reverberation echoes -Uncomfortable for patient and sometimes may need sedation -Intra- and inter-operator variability
Computed tomography	-Short acquisition time (suitable for unstable patients) -Wide availability -No contraindication in the presence of metallic devices -Full assessment of thoracoabdominal aorta -High spatial resolution -Optimal visualization of arterial wall calcification and endovascular stents	-Use of iodinated contrast (risk of contrast induced nephropathy and allergy) -Use of ionizing radiations (The tube parameters and the amount of contrast agents vary according to the type of scanner used. Consider the speed of scan acquisition and coverage to optimize image quality and reduce the amount of contrast agents)
Magnetic resonance imaging	-No radiation exposure -No iodinated contrast -Excellent evaluation of aortic wall -Dynamic assessment of flow -Gadolinium contrast media not mandatory -Not controindicated in pregnancy	-Prolonged scan time -Difficulty in monitoring acutely ill patients -Risk of nephrogenic systemic fibrosis with GFR < 30 mL/min/1.73 m^2^ (when gadolinium is used) -Poor assessment of arterial wall calcification -Signal loss within endovascular stent due lack of radiofrequency penetration -Less availability
Aortography	-Elevated accuracy -Necessary for endovascular procedures	-Invasive -Requires contrast medium -Entails radiation exposure

GFR, Glomerular Filtration Rate.

**Table 2:** Advantages and limitations of imaging techniques in the diagnosis of intramural hematoma.

Imaging Technique	Advantages	Limitations
Transesophageal echocardiography	-High-resolution images with direct observation of the aortic wall -Flow assessment with Doppler technique -Complication assessment (e.g., pericardial and pleural effusion and mediastinal haemorrhage)	-Difficult to visualize all segments of the aorta (e.g., ‘‘blind spot’’) -Semi-invasive technique -Operator dependent
Computed tomography	-Short acquisition time (suitable for unstable patients) -Wide availability -No contraindication in the presence of metallic devices -Full assessment of thoracoabdominal aorta -High spatial resolution	-Uses iodinated contrast (risk of contrast induced nephropathy and allergy) -Use of ionizing radiations -Difficult for the differential diagnosis with aortitis
Magnetic resonance imaging	-No radiation exposure -No iodinated contrast -Excellent evaluation of aortic wall and determination of the age of hematoma -Differential diagnosis between aortitis, thrombus, and dissection -Gadolinium contrast media not mandatory -Not controindicated in pregnancy	-Prolonged scan time -Difficulty in monitoring acutely ill patients -Risk of nephrogenic systemic fibrosis with GFR < 30 mL/min/1.73 m^2^ (when gadolinium is used)
Aortography	-Necessary for endovascular procedures	-Limited accuracy -Invasive -Requires contrast medium -Entails radiation exposure

GFR, Glomerular Filtration Rate.

**Table 3:** Advantages and limitations of imaging techniques in the diagnosis of penetrating aortic ulcer.

Imaging Technique	Advantages	Limitations
Transesophageal echocardiography	-High-resolution images with direct observation of the aortic wall -Differential diagnosis with ulcer-like projections	-Moderate diagnostic value -Semi-invasive technique -Operator dependent
Computed tomography	-Short acquisition time (suitable for unstable patients) -Wide availability -No contraindication in the presence of metallic devices -Full assessment of thoracoabdominal aorta -High spatial resolution	-Use of iodinated contrast (risk of contrast induced nephropathy and allergy) -Use of ionizing radiations
Magnetic resonance imaging	-No radiation exposure -No iodinated contrast -Excellent evaluation of aortic wall -Differential diagnosis between thrombus, IMH, ulcerated atherosclerotic plaque, and dissection -Gadolinium contrast media not mandatory -Not contraindicated in pregnancy	-Prolonged scan time -Difficulty in monitoring acutely ill patients -Risk of nephrogenic systemic fibrosis with GFR < 30 mL/min/1.73 m2 (when gadolinium is used)
Aortography	-Elevated accuracy -Necessary for endovascular procedures	-Invasive -Requires contrast medium -Entails radiation exposure

GFR, Glomerular Filtration Rate.

## Data Availability

No new data were generated or analysed in support of this research.

## References

[1] Mussa F.F., Horton J.D., Moridzadeh R., Nicholson J., Trimarchi S., Eagle K.A. (2016). Acute Aortic Dissection and Intramural Hematoma: A Systematic Review. JAMA.

[2] Goldstein S.A., Evangelista A., Abbara S., Arai A., Asch F.M., Badano L.P., Bolen M.A., Connolly H.M., Cuéllar-Calàbria H., Czerny M. (2015). Multimodality imaging of diseases of the thoracic aorta in adults: From the American Society of Echocardiography and the European Association of Cardiovascular Imaging: Endorsed by the Society of Cardiovascular Computed Tomography and Society for Cardiovascular Magnetic Resonance. J. Am. Soc. Echocardiogr..

[3] Evangelista A., Isselbacher E.M., Bossone E., Gleason T.G., Eusanio M.D., Sechtem U., Ehrlich M.P., Trimarchi S., Braverman A.C., Myrmel T. (2018). IRAD Investigators. Insights From the International Registry of Acute Aortic Dissection: A 20-Year Experience of Collaborative Clinical Research. Circulation.

[4] Bossone E., LaBounty T.M., Eagle K.A. (2018). Acute aortic syndromes: Diagnosis and management, an update. Eur. Heart J..

[5] Erbel R., Aboyans V., Boileau C., Bossone E., Bartolomeo R.D., Eggebrecht H., Evangelista A., Falk V., Frank H., Gaemperli O. (2014). ESC Committee for Practice Guidelines. 2014 ESC Guidelines on the diagnosis and treatment of aortic diseases: Document covering acute and chronic aortic diseases of the thoracic and abdominal aorta of the adult. The Task Force for the Diagnosis and Treatment of Aortic Diseases of the European Society of Cardiology (ESC). Eur. Heart J..

[6] Khandheria B.K., Tajik A.J., Taylor C.L., Safford R.E., Miller F.A., Stanson A.W., Sinak L.J., Oh J.K., Seward J.B. (1989). Aortic dissection: Review of value and limitations of two-dimensional echocardiography in a six-year experience. J. Am. Soc. Echocardiogr..

[7] Iliceto S., Ettorre G., Francioso G., Antonelli G., Biasco G., Rizzon P. (1984). Diagnosis of aneurysm of the thoracic aorta. Comparison between two non invasive techniques: Two-dimensional echocardiography and computed tomography. Eur. Heart J..

[8] Czerny M., Schmidli J., Adler S., van den Berg J.C., Bertoglio L., Carrel T., Chiesa R., Clough R.E., Eberle B., Etz C. (2019). EACTS/ESVS scientific document group. Current options and recommendations for the treatment of thoracic aortic pathologies involving the aortic arch: An expert consensus document of the European Association for Cardio-Thoracic surgery (EACTS) and the European Society for Vascular Surgery (ESVS). Eur. J. Cardiothorac. Surg..

[9] Evangelista A., Flachskampf F.A., Erbel R., Antonini-Canterin F., Vlachopoulos C., Rocchi G., European Association of Echocardiography (2010). Echocardiography in aortic diseases: EAE recommendations for clinical practice. Eur. J. Echocardiogr..

[10] Sobczyk D., Nycz K. (2015). Feasibility and accuracy of bedside transthoracic echocardiography in diagnosis of acute proximal aortic dissection. Cardiovasc. Ultrasound.

[11] Baliga R.R., Nienaber C.A., Bossone E., Oh J.K., Isselbacher E.M., Sechtem U., Fattori R., Raman S.V., Eagle K.A. (2014). The role of imaging in aortic dissection and related syndromes. JACC Cardiovasc. Imaging.

[12] Evangelista A., Avegliano G., Aguilar R., Cuellar H., Igual A., González-Alujas T., Rodríguez-Palomares J., Mahia P., García-Dorado D. (2010). Impact of contrast-enhanced echocardiography on the diagnostic algorithm of acute aortic dissection. Eur. Heart J..

[13] Flachskampf F.A., Wouters P.F., Edvardsen T., Evangelista A., Habib G., Hoffman P., Hoffmann R., Lancellotti P., Pepi M. (2014). Recommendations for transoesophageal echocardiography: EACVI update 2014. Eur. Heart J. Cardiovasc. Imaging.

[14] Pepi M., Campodonico J., Galli C., Tamborini G., Barbier P., Doria E., Maltagliati A., Alimento M., Spirito R. (2000). Rapid diagnosis and management of thoracic aortic dissection and intramural haematoma: A prospective study of advantages of multiplane vs. biplane transoesophageal echocardiography. Eur. J. Echocardiogr..

[15] Evangelista A., Garcia-del-Castillo H., Gonzalez-Alujas T., Dominguez-Oronoz R., Salas A., Permanyer-Miralda G., Soler-Soler J. (1996). Diagnosis of ascending aortic dissection by transesophageal echocardiography: Utility of M-mode in recognizing artifacts. J. Am. Coll. Cardiol..

[16] Evangelista A., Salas A., Ribera A., Ferreira-González I., Cuellar H., Pineda V., González-Alujas T., Bijnens B., Permanyer-Miralda G., Garcia-Dorado D. (2012). Long-term outcome of aortic dissection with patent false lumen: Predictive role of entry tear size and location. Circulation.

[17] Ueda T., Chin A., Petrovitch I., Fleischmann D. (2012). A pictorial review of acute aortic syndrome: Discriminating and overlapping features as revealed by ECG-gated multidetector-row CT angiography. Insights Imaging.

[18] Grewal S., Contrella B.N., Sherk W.M., Khaja M.S., Williams D.M. (2021). Endovascular Management of Malperfusion Syndromes in Aortic Dissection. Tech. Vasc. Interv. Radiol..

[19] McMahon M.A., Squirrell C.A. (2010). Multidetector CT of Aortic Dissection: A Pictorial Review. Radiographics.

[20] Carroll B.J., Schermerhorn M.L., Manning W.J. (2020). Imaging for acute aortic syndromes. Heart.

[21] Moore A.G., Eagle K.A., Bruckman D., Moon B.S., Malouf J.F., Fattori R., Evangelista A., Isselbacher E.M., Suzuki T., Nienaber C.A. (2002). Choice of computed tomography, transesophageal echocardiography, magnetic resonance imaging, and aortography in acute aortic dissection: International Registry of Acute Aortic Dissection (IRAD). Am. J. Cardiol..

[22] Nienaber C.A. (2013). The role of imaging in acute aortic syndromes. Eur. Heart J. Cardiovasc. Imaging.

[23] Sebastià C., Pallisa E., Quiroga S., Alvarez-Castells A., Dominguez R., Evangelista A. (1999). Aortic dissection: Diagnosis and follow-up with helical CT. Radiographics.

[24] Restrepo C.S., Lemos D.F., Lemos J.A., Velasquez E., Diethelm L., Ovella T.A., Martinez S., Carrillo J., Moncada R., Klein J.S. (2007). Imaging findings in cardiac tamponade with emphasis on CT. Radiographics.

[25] Murillo H., Molvin L., Chin A.S., Fleischmann D. (2021). Aortic Dissection and Other Acute Aortic Syndromes: Diagnostic Imaging Findings from Acute to Chronic Longitudinal Progression. Radiographics.

[26] Shiga T., Wajima Z., Apfel C.C., Inoue T., Ohe Y. (2006). Diagnostic accuracy of transesophageal echocardiography, helical computed tomography, and magnetic resonance imaging for suspected thoracic aortic dissection: Systematic review and meta-analysis. Arch. Intern. Med..

[27] Wang G.X., Hedgire S.S., Le T.Q., Sonis J.D., Yun B.J., Lev M.H., Raja A.S., Prabhakar A.M. (2017). MR angiography can guide ED management of suspected acute aortic dissection. Am. J. Emerg. Med..

[28] Evangelista A., Carro A., Moral S., Teixido-Tura G., Rodríguez-Palomares J.F., Cuéllar H., García-Dorado D. (2013). Imaging modalities for the early diagnosis of acute aortic syndrome. Nat. Rev. Cardiol..

[29] Sakuma H., Bourne M.W., O’Sullivan M., Merrick S.H., Ullyot D.J., Chatterjee K., Shimakawa A., Foo T.K., Higgins C.B. (1996). Evaluation of thoracic aortic dissection using breath-holding cine MRI. J. Comput. Assist. Tomogr..

[30] Powell A.J., Maier S.E., Chung T., Geva T. (2000). Phase-velocity cine magnetic resonance imaging measurement of pulsatile blood flow in children and young adults: In vitro and in vivo validation. Pediatr. Cardiol..

[31] Chang J.M., Friese K., Caputo G.R., Kondo C., Higgins C.B. (1991). MR measurement of blood flow in the true and false channel in chronic aortic dissection. J. Comput. Assist. Tomogr..

[32] Silverman J.M., Raissi S., Tyszka J.M., Trento A., Herfkens R.J. (2000). Phase-contrast cine MR angiography detection of thoracic aortic dissection. Int. J. Card. Imaging.

[33] Pereles F.S., McCarthy R.M., Baskaran V., Carr J.C., Kapoor V., Krupinski E.A., Finn J.P. (2002). Thoracic aortic dissection and aneurysm: Evaluation with nonenhanced true FISP MR angiography in less than 4 minutes. Radiology.

[34] Gebker R., Gomaa O., Schnackenburg B., Rebakowski J., Fleck E., Nagel E. (2007). Comparison of different MRI techniques for the assessment of thoracic aortic pathology: 3D contrast enhanced MR angiography, turbo spin echo and balanced steady state free precession. Int. J. Cardiovasc. Imaging.

[35] Kinner S., Eggebrecht H., Maderwald S., Barkhausen J., Ladd S.C., Quick H.H., Hunold P., Vogt F.M. (2015). Dynamic MR angiography in acute aortic dissection. J. Magn. Reson. Imaging.

[36] Veger H.T.C., Pasveer E.H., Westenberg J.J.M., Wever J.J., van Eps R.G.S. (2021). Wall Shear Stress Assessment of the False Lumen in Acute Type B Aortic Dissection Visualized by 4-Dimensional Flow Magnetic Resonance Imaging: An Ex-Vivo Study. Vasc. Endovasc. Surg..

[37] Chen C.W., Tseng Y.H., Lin C.C., Kao C.C., Wong M.Y., Ting H., Huang Y.K. (2021). Aortic dissection assessment by 4D phase-contrast MRI with hemodynamic parameters: The impact of stent type. Quant. Imaging Med. Surg..

[38] Khandheria B.K. (1993). Aortic dissection. The last frontier. Circulation.

[39] Bansal R.C., Chandrasekaran K., Ayala K., Smith D.C. (1995). Frequency and explanation of false negative diagnosis of aortic dissection by aortography and transesophageal echocardiography. J. Am. Coll. Cardiol..

[40] Cecconi M., Chirillo F., Costantini C., Iacobone G., Lopez E., Zanoli R., Gili A., Moretti S., Manfrin M., Münch C. (2012). The role of transthoracic echocardiography in the diagnosis and management of acute type A aortic syndrome. Am. Heart J..

[41] Song J.K. (2004). Diagnosis of aortic intramural haematoma. Heart.

[42] Brown J.A., Arnaoutakis G.J., Kilic A., Gleason T.G., Aranda-Michel E., Sultan I. (2020). Current trends in the management of acute type A aortic intramural hematoma. J. Card. Surg..

[43] Ionescu A.A., Vinereanu D., Wood A., Fraser A.G. (1998). Periaortic fat pad mimicking an intramural hematoma of the thoracic aorta: Lessons for transesophageal echocardiography. J. Am. Soc. Echocardiogr..

[44] Chao C.P., Walker T.G., Kalva S.P. (2009). Natural history and CT appearances of aortic intramural hematoma. Radiographics.

[45] Harris K.M., Braverman A.C., Eagle K.A., Woznicki E.M., Pyeritz R.E., Myrmel T., Peterson M.D., Voehringer M., Fattori R., Januzzi J.L. (2012). Acute aortic intramural hematoma: An analysis from the International Registry of Acute Aortic Dissection. Circulation.

[46] Herrán F.L., Bang T.J., Restauri N., Suby-Long T., Alvarez Gómez D.I., Sachs P.B., Vargas D. (2018). CT imaging of complications of aortic intramural hematoma: A pictorial essay. Diagn. Interv. Radiol..

[47] Nienaber C.A., von Kodolitsch Y., Petersen B., Loose R., Helmchen U., Haverich A., Spielmann R.P. (1995). Intramural hemorrhage of the thoracic aorta. Diagnostic and therapeutic implications. Circulation.

[48] Yamada T., Tada S., Harada J. (1988). Aortic dissection without intimal rupture: Diagnosis with MR imaging and CT. Radiology.

[49] Murray J.G., Manisali M., Flamm S.D., VanDyke C.W., Lieber M.L., Lytle B.W., White R.D. (1997). Intramural hematoma of the thoracic aorta: MR image findings and their prognostic implications. Radiology.

[50] Schwein A., Khan M., Bennett M., Chakfé N., Lumsden A.B., Bismuth J., Shah D.J. (2019). Proposed Magnetic Resonance Imaging Criteria to Diagnose Intramural Haematoma and to Predict Aortic Healing after Acute Type B Aortic Syndrome. Eur. J. Vasc. Endovasc. Surg..

[51] Buckley O., Rybicki F.J., Gerson D.S., Huether C., Prior R.F., Powers S.L., Ersoy H. (2010). Imaging features of intramural hematoma of the aorta. Int. J. Cardiovasc. Imaging.

[52] Yamada I., Numano F., Suzuki S. (1993). Takayasu arteritis: Evaluation with MR imaging. Radiology.

[53] Braverman A.C. (1994). Penetrating atherosclerotic ulcers of the aorta. Curr. Opin. Cardiol..

[54] Nathan D.P., Boonn W., Lai E., Wang G.J., Desai N., Woo E.Y., Fairman R.M., Jackson B.M. (2012). Presentation, complications, and natural history of penetrating atherosclerotic ulcer disease. J. Vasc. Surg..

[55] Oderich G.S., Kärkkäinen J.M., Reed N.R., Tenorio E.R., Sandri G.A. (2019). Penetrating Aortic Ulcer and Intramural Hematoma. Cardiovasc. Interv. Radiol..

[56] Evangelista A., Moral S., Ballesteros E., Castillo-Gandia A. (2020). Beyond the term penetrating aortic ulcer: A morphologic descriptor covering a constellation of entities with different prognoses. Prog. Cardiovasc. Dis..

[57] Sorber R., Hicks C.W. (2022). Diagnosis and Management of Acute Aortic Syndromes: Dissection, Penetrating Aortic Ulcer, and Intramural Hematoma. Curr. Cardiol. Rep..

[58] Macura K.J., Szarf G., Fishman E.K., Bluemke D.A. (2003). Role of computed tomography and magnetic resonance imaging in assessment of acute aortic syndromes. Semin Ultrasound CT MR.

[59] Yucel E.K., Steinberg F.L., Egglin T.K., Geller S.C., Waltman A.C., Athanasoulis C.A. (1990). Penetrating aortic ulcers: Diagnosis with MR imaging. Radiology.

[60] Stanson A.W., Kazmier F.J., Hollier L.H., Edwards W.D., Pairolero P.C., Sheedy P.F., Joyce J.W., Johnson M.C. (1986). Penetrating atherosclerotic ulcers of the thoracic aorta: Natural history and clinicopathologic correlations. Ann. Vasc. Surg..

[61] Cooke J.P., Kazmier F.J., Orszulak T.A. (1988). The penetrating aortic ulcer: Pathologic manifestations, diagnosis, and management. Mayo Clinic Proceedings.

[62] Bhave N.M., Nienaber C.A., Clough R.E., Eagle K.A. (2018). Multimodality Imaging of Thoracic Aortic Diseases in Adults. JACC Cardiovasc. Imaging.

[63] Nazerian P., Pivetta E., Veglia S., Cavigli E., Mueller C., de Matos Soeiro A., Leidel B.A., Lupia E., Rutigliano C., Wussler D. (2019). ADvISED Investigators. Integrated Use of Conventional Chest Radiography Cannot Rule Out Acute Aortic Syndromes in Emergency Department Patients at Low Clinical Probability. Acad. Emerg. Med..

[64] Bima P., Pivetta E., Nazerian P., Toyofuku M., Gorla R., Bossone E., Erbel R., Lupia E., Morello F. (2020). Systematic Review of Aortic Dissection Detection Risk Score Plus D-dimer for Diagnostic Rule-out Of Suspected Acute Aortic Syndromes. Acad. Emerg. Med..

[65] Morello F., Bima P., Pivetta E., Santoro M., Catini E., Casanova B., Leidel B.A., de Matos Soeiro A., Nestelberger T., Mueller C. (2021). Development and Validation of a Simplified Probability Assessment Score Integrated with Age-Adjusted d-Dimer for Diagnosis of Acute Aortic Syndromes. J. Am. Heart Assoc..

[66] Nazerian P., Mueller C., Soeiro A.M., Leidel B.A., Salvadeo S.A.T., Giachino F., Vanni S., Grimm K., Oliveira M.T., Pivetta E. (2018). ADvISED Investigators. Diagnostic Accuracy of the Aortic Dissection Detection Risk Score Plus D-Dimer for Acute Aortic Syndromes: The ADvISED Prospective Multicenter Study. Circulation.

[67] Pape L.A., Awais M., Woznicki E.M., Suzuki T., Trimarchi S., Evangelista A., Myrmel T., Larsen M., Harris K.M. (2015). Presentation, Diagnosis, and Outcomes of Acute Aortic Dissection: 17-Year Trends From the International Registry of Acute Aortic Dissection. J. Am. Coll. Cardiol..

[68] Sonaglioni A., Lombardo M., Rigamonti E., Nicolosi G.L., Trevisan R., Zompatori M., Anzà C. (2021). An unusual case of painless type A aortic dissection. J. Clin. Ultrasound.

[69] Penco M., Paparoni S., Dagianti A., Fusilli C., Vitarelli A., De Remigis F., Mazzola A., Di Luzio V., Gregorini R., D’Eusanio G. (2000). Usefulness of transesophageal echocardiography in the assessment of aortic dissection. Am. J. Cardiol..

[70] Smith A.D., Schoenhagen P. (2008). CT imaging for acute aortic syndrome. Clevel. Clin. J. Med..

[71] Davenport M.S., Perazella M.A., Yee J., Dillman J.R., Fine D., McDonald R.J., Rodby R.A., Wang C.L., Weinreb J.C. (2020). Use of Intravenous Iodinated Contrast Media in Patients with Kidney Disease: Consensus Statements from the American College of Radiology and the National Kidney Foundation. Kidney Med..

[72] Lunyera J., Mohottige D., Alexopoulos A.S., Campbell H., Cameron C.B., Sagalla N., Amrhein T.J., Crowley M.J., Dietch J.R., Gordon A.M. (2020). Risk for Nephrogenic Systemic Fibrosis After Exposure to Newer Gadolinium Agents: A Systematic Review. Ann. Intern. Med..

[73] Weinreb J.C., Rodby R.A., Yee J., Wang C.L., Fine D., McDonald R.J., Perazella M.A., Dillman J.R., Davenport M.S. (2021). Use of intravenous gadolinium-based contrast media in patients with kidney disease: Consensus statements from the American College of Radiology and the National Kidney Foundation. Radiology.

[74] Lohan D.G., Krishnam M., Saleh R., Tomasian A., Finn J.P. (2008). MR imaging of the thoracic aorta. Magn. Reson. Imaging Clin. N. Am..

[75] Isselbacher E.M., Preventza O., Hamilton Black Iii J., Augoustides J.G., Beck A.W., Bolen M.A., Braverman A.C., Bray B.E., Brown-Zimmerman M.M., Chen. E.P. (2022). 2022 ACC/AHA Guideline for the Diagnosis and Management of Aortic Disease: A Report of the American Heart Association/American College of Cardiology Joint Committee on Clinical Practice Guidelines. J. Am. Coll. Cardiol..

[76] Sievers H.H., Richardt D., Diwoky M., Auer C., Bucsky B., Nasseri B., Klotz S. (2018). Survival and reoperation after valve-sparing root replacement and root repair in acute type A dissection. J. Thorac. Cardiovasc. Surg..

[77] Giles K.A., Beck A.W., Lala S., Patterson S., Back M., Fatima J., Arnaoutakis D.J., Arnaoutakis G.J., Beaver T.M., Berceli S.A. (2019). Implications of secondary aortic intervention after thoracic endovascular aortic repair for acute and chronic type B dissection. J. Vasc. Surg..

[78] Wang H., Wagner M., Benrashid E., Keenan J., Wang A., Ranney D., Yerokun B., Gaca J.G., McCann R.L., Hughes G.C. (2017). Outcomes of Reoperation After Acute Type A Aortic Dissection: Implications for Index Repair Strategy. J. Am. Heart Assoc..

[79] Yang L., Zhang Q.Y., Wang X.Z., Zhao X., Liu X.Z., Wang P., Jing Q.M., Han Y.L. (2020). Long-Term Imaging Evolution and Clinical Prognosis Among Patients with Acute Penetrating Aortic Ulcers: A Retrospective Observational Study. J. Am. Heart Assoc..

